# New Omnidirectional Sensor Based on Open-Source Software and Hardware for Tracking and Backtracking of Dual-Axis Solar Trackers in Photovoltaic Plants

**DOI:** 10.3390/s21030726

**Published:** 2021-01-21

**Authors:** Francisco J. Gómez-Uceda, José Ramirez-Faz, Marta Varo-Martinez, Luis Manuel Fernández-Ahumada

**Affiliations:** 1 Department of Mechanics, Campus of Rabanales, University of Cordoba, 14071 Cordoba, Spain; fjgomez@uco.es; 2 Department of Electrical Engineering and Automatics, Campus of Rabanales, University of Cordoba, 14071 Cordoba, Spain; jramirez@uco.es (J.R.-F.); lmfernandez@uco.es (L.M.F.-A.); 3 Department of Applied Physics, Radiology and Physical Medicine, Campus of Rabanales, University of Cordoba, 14071 Cordoba, Spain

**Keywords:** free and open-source hardware (FOSH), sun position sensor, omnidirectional sensor, solar trackers, PV plants, backtracking

## Abstract

In this work, an omnidirectional sensor that enables identification of the direction of the celestial sphere with maximum solar irradiance is presented. The sensor, based on instantaneous measurements, functions as a position server for dual-axis solar trackers in photovoltaic plants. The proposed device has been developed with free software and hardware, which makes it a pioneering solution because it is open and accessible as well as capable of being improved by the scientific community, thereby contributing to the rapid advancement of technology. In addition, the device includes an algorithm developed ex professo that makes it possible to predetermine the regions of the celestial sphere for which, according to the geometric characteristics of the PV plant, there would be shading between the panels. In this way, solar trackers do not have to locate the Sun’s position at all times according to astronomical models, while taking into account factors such as shadows or cloudiness that also affect levels of incident irradiance on solar collectors. Therefore, with this device, it is possible to provide photovoltaic plants with dual-axis solar tracking with a low-cost device that helps to optimise the trajectory of the trackers and, consequently, their radiative capture and energy production.

## 1. Introduction

The industrial and technological development that society has undergone, as well as the increase in the population worldwide, has led to a growing demand for energy [1,2]. Satisfying this increase in energy demand only by means of traditional methods of energy production based on fossil and nuclear resources entails serious environmental problems that endanger the sustainability of the Earth, such as pollution and climate change [3,4,5]. In response, the scientific community has highlighted the importance of enhancing the role of renewable energies in the energy models of both developed and developing countries [1,6,7]. In fact, the number of journals and papers related to renewable energies has experienced a remarkable growth [8], which shows the increasing researchers’ awareness of the need to contribute to the improvement and the progress of this field of science and its beneficial impact on the challenges of current society.

Among these possible renewable energy sources, solar energy plays a fundamental role [9,10,11] since, as stated by Kannan and Vakeesan [3], it is an abundant source of energy that, being properly exploited, could be enough to satisfy world energy demand. Furthermore, it is available all over the planet, its use has no negative impact on the environment and it is a technology that is easily usable at all levels (industrial, domestic, etc.). The technological improvements achieved in recent years have allowed to reduce the production cost of PV energy to values competitive with those of the energy supplied by the grid [12]. As a result, the presence of PV technologies in the energy market has experienced a significant growth [13]. However, in order to continue promoting this expansion, it is necessary to continue researching into new solutions that will maintain their growing development and technological progress [12].

### 1.1. Literature Review on Solar Tracking

Among the solar energy technologies, photovoltaic (PV) is undergoing a remarkable boom due to its simplicity and low cost, as well as the significantly technological enhancements that it has been experiencing. As a consequence, it is becoming a promising source of electricity generation [14]. However, despite its rapid technological evolution, there is still plenty of room for optimisation in the efficiency of the management of photovoltaic installations, as well as in the configuration of its design, which would lead to a potential increase in its development.

One possible line of technological improvement of PV that has been worked on for decades is solar tracking [9]. It tries to alleviate the negative effects of the high variability of the solar resource, both in time as well as in space, by reorienting the PV panels towards possible directions that increase solar irradiance collection. In order to do so, solar trackers are very useful both in large PV plants connected to the grid and in small domestic installations in which the space available for the installation of the panels is often reduced and, as a consequence, it is necessary to increase the energy generated per square metre of collecting surface [15].

There is a traditional classification of trackers based on the degrees of freedom of the tracking movement according to which they can be categorised into single-axis trackers and dual-axis trackers. The former is characterised by modifying the orientation of the collector plane by turning around a single fixed axis. The latter are characterised by a movement of its plane through the rotation of a system composed of two fixed axes, which allows it to orient itself in any possible direction in the celestial sphere [16]. Although dual-axis trackers are more expensive and require more work to implement and maintain than single-axis trackers, they offer better performance [17,18,19]. In fact, although some authors affirm that dual-axis monitoring systems have no future due to their complexity and high cost [20,21], Eldin et al. [14] suggest that, at present, this type of technology is widespread throughout the world and that multiple research is being developed to improve both the technology and its efficiency/cost ratio, so that its energy production exceeds and compensates for the costs of the installation and maintenance as well as the energy consumption used in the movement of the trackers.

Another possible classification of solar trackers is the one based on the mechanism that enables monitoring. Thus, on the one hand, there are passive trackers that do not use mechanical devices for movement. To the contrary, in most cases, they are composed of a pair of actuators, filled with expandable gas, which in the case of imbalance, are levelled with equal lighting by means of thermal expansion [22]. In comparison, active solar trackers use motors commonly governed by control signals for movement in search of the position of the Sun, which are very precise devices except on very cloudy days [23].

Finally, depending on the tracking control strategy, a distinction is made between trackers in which the movement, both in azimuth and elevation, is governed by mathematical models (in open loop) and those in which the system feeds back through irradiance sensors (closed loop).

Various literature review works have systematically collected the data obtained by different solar trackers developed by the scientific community, finding that the energy produced by a PV system with tracking is always greater than that of a system without it [9,10], except on spring or summer days with great cloudiness [17,24]. More specifically, Eldin et al. [14] carried out a study on the convenience of monitoring systems depending on the climatic conditions of the place and verified that the output power of photovoltaic panels with solar monitoring depends on environmental conditions. Thus, while in cold regions with a high incidence of cloudiness, monitoring strategies are profitable for maximising the power of photovoltaic panels, in places with very hot and sunny climates, they are not, due to the negative influence of overheating on performance of photovoltaic panels. Likewise, some authors have analysed the improvements in energy production of PV systems with solar tracking depending on the type of technology used and the latitude of the study site [25,26,27]. Thus, it has been shown that, in general, the higher the latitude, the better the monitoring efficiency is achieved, reaching improvements of up to 57% [23].

Similarly, with regard to grid-connected PV installations, a recent study [28] has analysed, from a techno-economic-environmental point of view, the use of different solar tracking systems to maximise the photovoltaic power generation in residential solar installations connected to the grid in eight regions of Iran with diverse climates. Based on the study carried out, they found that the dual-axis monitoring system is the most efficient (32% average increase in energy production compared to an installation without monitoring), while the vertical single-axis monitoring system is the most profitable (23% increase in energy production compared to a nonmonitored installation with only 1.6% increase in energy cost). In general terms, the study concludes that the use of the solar tracking system in residential installations connected to the grid significantly reduces the number of panels needed, but this reduction in size is not always profitable due to the high cost of the monitoring units. However, the profitability of the installation increases significantly in all cases when the sale of electricity to the grid is allowed.

As far as the monitoring strategy is concerned, the most frequent in the literature is that based on solar astronomical movement, which aims to minimise the angle of incidence θ between the solar rays and the normal to the capture surface. According to this astronomical tracking strategy, various works [29,30,31,32,33,34] show prediction models of incident irradiance on the plane of trackers of both single and dual axes, with a degree of accuracy for solar location in the celestial sphere to the order of mrad [35,36,37]. The models used for astronomical tracking have traditionally been based on spherical trigonometry [31]. However, recently, a new paradigm using vector algebra to define the solar movement and that of the trackers can be found in the literature [38,39,40,41,42,43,44,45]. For this, these models use the solar vector $\vec{s}$, which is a unit vector that is directed to the centre of the solar disk. Its expression in different coordinate systems and the use of the definition of scalar and vector product enable the deduction of the entire system of astronomical relationships that govern the movement of the solar trackers [46].

Furthermore, as previously mentioned, the astronomical tracking strategy seeks the optimisation of the direct component of solar irradiance. Consequently, it is adapted to solar concentrators that are based on the use of this component, but not to flat PV collectors in which the remaining components of irradiance (diffuse and reflected) are also used. Thus, on days when the solar disk is not visible and direct irradiance does not reach the collectors, the efficiency of this monitoring strategy is not satisfactory [23,30] and the capture of the collectors is less than that which would be obtained on a horizontal flat surface. Despite this, it is difficult to find references that determine models for solar tracking on these types of days, so it is necessary to continue developing mathematical equations that also take into account the diffuse and reflected components when trying to maximise radiative collection as part of solar tracking strategy. 

On the other hand, the energy reduction caused by shading is particularly significant for PV installations. In addition, the shaded cells become overheated, which may lead to a fast degradation of the modules. Backtracking is applied to prevent the inter-shading of collectors. This technique consists of shifting the collectors to positions where shadows no longer appear [26,32,47]. Combining these two requirements (optimising global irradiance and performing backtracking) leads to a differentiation within the dedicated and specific tracking strategy for PV plants, studied further in this article.

In this line of work, a novel solar tracking strategy with back-tracking has been proposed to optimise the capture of solar irradiance at all times while avoiding inter-shading between collectors in PV plants with dual-axis tracking [39,40,41]. In this study, based on empirical models for the characterisation of the hemispheric distribution of irradiance, the authors quantify the increases in solar incidence on collectors at a higher value than 2%. In order to implement this strategy in existing facilities, the device presented in this article is developed and built.

Likewise, other authors [17,48,49,50] have implemented tracking systems with sensors that follow the position of the Sun with great precision and that have the advantages of easy implementation, simple design, low cost and a high level of adaptability. However, it is necessary to continue advancing in the search for tracking strategies that enable constant identification of the direction of the celestial sphere in which solar irradiance is maximum in a simple way regarding the hardware and software necessary for its implementation and that does not imply an increase in the cost of the technology, either in implementation or maintenance.

### 1.2. Literature Review on Free and Open-Source Hardware and Software Applied to PV Energy

Despite the great progress that new technologies have experienced in recent decades, the energy supply network based on traditional technologies has not evolved at the same rates [5]. However, this is different in the case of renewable energies. In that sense, it is increasingly common to find in the literature proposals based on free hardware in the field of photovoltaic solar energy, in general, and in solar tracking, in particular. Thus, for example, the use of microcontrollers (many based on free hardware) in the implementation of various photovoltaic tracking strategies presents an important competitive advantage at an economic level compared to control based on traditional PLCs [51]. In general terms, with the use of technologies based on free hardware in the field of solar PV energy, not only are lower costs sought, but it is also intended that the results and yields obtained are similar to or better than those achieved by commercial solutions [52]. In this sense, as it is a free hardware system, it can be shared among the scientific community and can be edited and improved by different experts [53]. Another advantage is the fact that the application of the devices shows a wide range of possibilities both at the level of capture (irradiance, temperature, and humidity) and control of the complex processes in which it works [54]. In addition, the possibility of safely, quickly and easily storing the huge amount of data generated by any photovoltaic installation is an important milestone in working with free hardware devices [55].

Among some of the devices found in the literature is the one by Gutierrez et al. [15] that presents a single-axis solar tracker for the integration of buildings controlled with an open-loop control strategy implemented through Arduino and IoT. This makes it a low-cost device with a flexible implementation and applicable anywhere in the world. A new electronic sensor based on free hardware has also been developed, validated and patented to measure radiation and global radiation on the horizontal surface [56]. The device is characterised by high precision and the technologies used in its implementation (Arduino and IoT) make it a low-cost device with a high level of connectivity and ubiquity, which is why it is easily applicable to the monitoring and control of any PV plant and, especially, to “smart-grid” solutions. Paredes-Parra et al. [57] have also developed a low-cost and open-source system, based on IoT and LoRa, which allows remote monitoring and real-time operation of a PV plant and, therefore, facilitating maintenance and supervision tasks. Similarly, Pereira et al. [58] have developed a new multi-user remote data acquisition and transmission system, based on Raspberry Pi and IoT technology, to monitor a photovoltaic plant in real time. Therefore, it can be affirmed that the relationship established in the different levels of aggregation of solar energy (generation, smart grids and integration) is a field in which the use of the aforementioned technologies finds an interesting space due to the versatility shown [59,60].

In accordance with all the above and combining the two lines of work presented, this paper describes a sensor that uses an onmidirectional solar tracker, based on Free and open-source hardware (FOSH), which acts as a server of position for dual-axis PV trackers, identifying at each instant in time, the optimal orientation of the PV panels from instantaneous irradiance measurements. With this, the solar trackers do not have to search for the position of the sun using algorithms based on solar geometry while taking into account other conditions (cloudiness, shading between panels, etc.) that also influence the irradiance received by the capturing surfaces. In this way, it is possible to provide PV plants with dual-axis solar tracking with a low-cost device that helps to optimise the trajectory of its trackers and, consequently, its radiative capture and energy production.

Following this introduction, the remainder of the article is organised as follows: in the next section, Section 2, the proposed design and the algorithms implemented in the device are outlined; Section 3 presents how the system was tested for a PV plant in Peñarroya (Spain) and discusses the results. In Section 4, conclusions are drawn based on the work developed.

## 2. Proposed Design

To achieve the objective described above, the device presented makes a scan of the celestial sphere, during which the incident irradiance measurement is carried out in order to determine the orientation for which this magnitude is maximum. However, as a novelty, the device incorporates a dichotomous algorithm, designed by the authors [39,40] that, prior to the scanning of the celestial sphere, identifies those orientations of the solar trackers for which there would be inter-shading between the collectors. With this, the proposed device restricts the search field for the orientation of maximum irradiance to the set of spatial directions in which there is no inter-shading, which in practice implies a backtracking strategy. Once the direction of maximum irradiance has been identified, the azimuth $\left(\gamma \right)$ and elevation $\left(\alpha \right)$ angles corresponding to it, stored in the device that acts as a position server, are made available to the solar trackers of the PV installation for their orientation towards the position of maximum capture.

The technological solution presented consists of a pan-tilt type orientation mechanism that allows the positioning of an irradiance sensor in any direction of the celestial sphere, characterised by its azimuth $\left(\gamma \right)$ and its elevation $\left(\alpha \right)$, as well as an irradiance measurement and control system in real time. The mechanism is controlled by a microprocessor that is also in charge of carrying out the irradiance readings and their transmission to the solar servers. For its operation, the necessary algorithms have been developed and implemented to adjust the movements of the solar trackers so that optimal energy production is achieved. Likewise, the complete architecture of the device has been developed, based on Free and Open-Source hardware (FOSH) and a simple control system with functionalities associated with IoT technologies. All of this makes the device an economically competitive tilt and azimuth server, capable of integrating into dual-axis photovoltaic installations and favouring the optimisation of its energy production. This previous dichotomous algorithm for the detection of inter-shading as well as the electronic and mechanical design of the device is described below.

### 2.1. Algorithm for the Detection of Inter-Shading between Collectors

As mentioned above, the device includes a simple and programmable algorithm in 8-bit AVR RISC microprocessors that, taking into account the characteristics of the PV installation, enables one to know whether a certain orientation $\left(\gamma ,\;\alpha \right)$ of the collectors would imply the partial inter-shading between them for a certain Julian day $\left(d_{j}\right)$ and a specific solar hour $\left(t\right)$ prior to the scanning of the celestial sphere. This algorithm is supported by a novel tracking strategy developed by the authors and the one that is based on Minkowski algebra [39,40].

**Figure 1:**
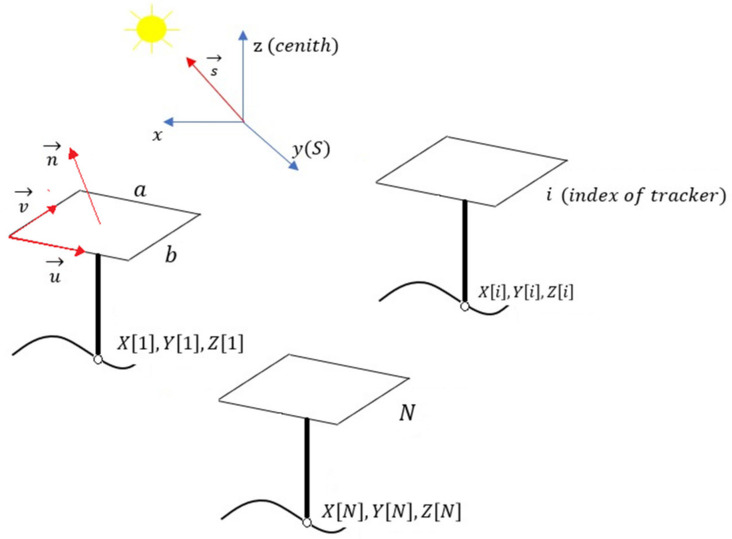
Geometric elements necessary to determine the existence of inter-shading according to the algorithm of Fernandez-Ahumada et al. [39,40].

This procedure can be understood as a Boolean function dependent on ($\gamma ,\;\alpha ,\;d_{j},t)$ in which the result “TRUE” implies the existence of inter-shading and “FALSE,” the absence. As auxiliary information, this function requires:

1. The width $\left(a\right)$ and height $\left(b\right)$ of the solar collectors.
2. The set of Cartesian coordinates$\;\left(x,y,z\right)$ of the base of each solar tracker, using as a reference system a local coordinate system in which the Ox axis goes to the West, the Oy to the South and the Oz to the point Zenith. This information is structured by three arrays$\;x [i],\;y [i],\;z [i]$ in which i is the index assigned to each solar tracker (Figure 1), so that 1<i<N is verified, where N is the number of trackers in the installation.
3. The solar vector or unit vector that points to the solar disk at each instant of time that, in the reference system considered, is given by the Equation (1):
  (1) $$\begin{array}{c} \vec{s}=s_{x}\vec{i}+s_{y}\vec{j}+s_{z}\vec{k}=\\ =\sin\Omega t\cos\delta \vec{i}+\\ +\left(\cos\Omega t\cos\delta \sin\varphi -\sin\delta \cos\varphi \right)\vec{j}+\\ +\left(\cos\Omega t\cos\delta \cos\varphi +\sin\delta \sin\varphi \right)\vec{k}, \end{array}$$
  where φ is the latitude, Ωt is the hourly angle, defined as the product of the Earth rotation speed (Ω=2π/24rad/h) and the time elapsed since solar noon, and δ is the solar declination given by Equation (2), being Γ and auxiliary angle dependent on the Julian day according to Equation (3)
  (2) $$\begin{array}{c} \delta \left(rad\right)=[0.006918-0.399912\cos\left(\Gamma \right)+\\ +0.070257\sin\left(\Gamma \right)-0.006758\cos\left(2\Gamma \right)+\\ +0.000907\sin\left(2\Gamma \right)-0.002697\cos\left(3\Gamma \right)+\\ +0.00148\sin\left(3\Gamma \right)], \end{array}$$
  (3) $$\Gamma \left(rad\right)=\frac{2\pi (d_{j}-1)}{365}.$$
4. The unit vector $\vec{n}$, which indicates the direction towards which the solar trackers are oriented, being perpendicular to the collectors, and which is given by the Equation (4).
  (4) $$\vec{n}=\cos\alpha \cdot \sin\gamma \vec{i}+\cos\alpha \cdot \cos\gamma \vec{j}+\sin\alpha \vec{k},$$
5. The unit vectors $\vec{u}$ y $\vec{v}$ included in the collector plane, where $\vec{u}$ is horizontal (Equation (5)) and $\vec{v}$ (Equation (6)) perpendicular to $\vec{u}$.
  (5) $$\vec{u}=-\cos\gamma \vec{i}+\sin\gamma \vec{j},$$
  (6) $$\vec{v}=\sin\alpha \cdot \cos\gamma \vec{i}-\sin\alpha \cdot \cos\gamma \vec{j}+\cos\alpha \vec{k}.$$

**Figure 2:**
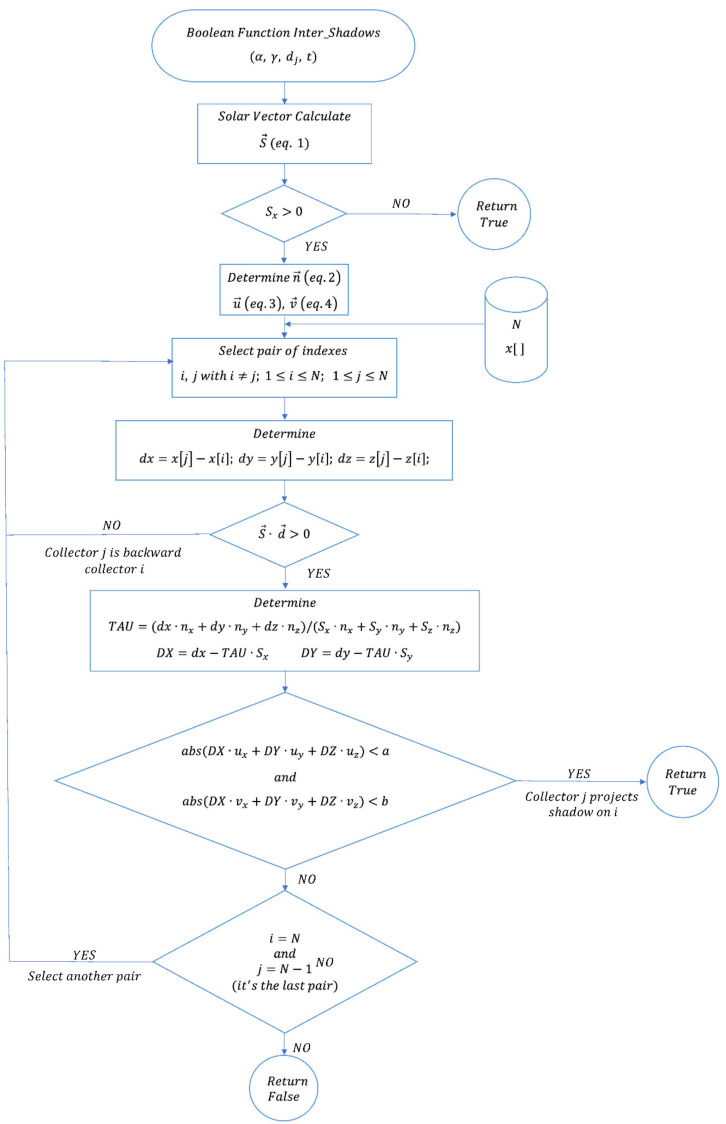
Flow chart of the designed procedure.

Figure 2 shows the flow chart of the designed and implemented procedure.

### 2.2. Design of the Proposed Technological Solution

**Figure 3:**
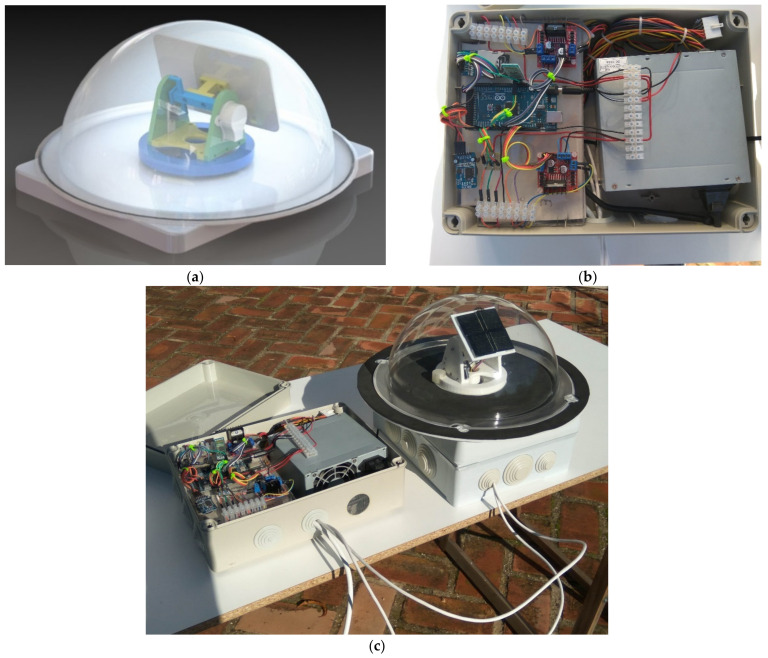
(**a**) Design of the system, (**b**) electronical components, and (**c**) photography of the prototype.

The mechanical design of the proposed device has been resolved by means of a flat surface with two degrees of freedom (Figure 3). The manufacture has been carried out by means of additive printing on acrylonitrile butadiene styrene (ABS) filament, a thermoplastic polymer with good properties with regard to distortion and softening temperatures, 96 and 93 °C, respectively. The whole set remains inside a transparent methacrylate dome.

**Figure 4:**
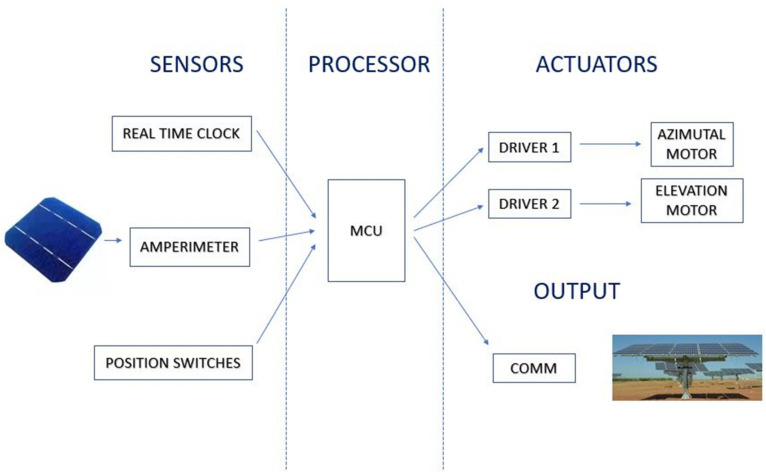
Scheme of principle of the proposed system.

Figure 4 schematically shows the concept of the electronic design of the system in which four blocks are distinguished: sensors, processing, actuators and communications.

- Sensors: On the one hand, the system includes a sensor system whose purpose is to know the solar time corresponding to orientations that are not allowed because they cause inter-shading between the collectors. For this, among the different options to obtain the time (internal clock of the microcontroller, time server or external RTC module), in this prototype, a DS1307 real-time clock has been chosen, with autonomous power supply by means of a CR2025 battery. Likewise, for the irradiance measurement, a calibrated photovoltaic cell of the Fadisol C-0121 type has been used that provides a linear current output with respect to irradiance, comprised between 36 mA for 125 W/m^2^ and 288 mA for 1000 W/m^2^. The measurement of the intensity of the electric current provided by these short-circuited photovoltaic cells is measured by means of an INA219 module, consisting of a shunt equipped with a 12-bit analog-digital converter and I2C output. In this way, adjusting the gain in the module configuration, an accuracy of 0.1 mA and a maximum intensity of 400 mA are obtained. Finally, an initialisation of the azimuth and elevation position has been provided, using two mechanical micro-switches that indicate the zero relative position to the microcontroller.
- Processing: In the philosophy of this work, several alternatives for processing have been evaluated, opting for a TTGO ESP32 Lora development board. The ESP32 microcontroller integrates analog and digital inputs and outputs, as well as various communication interfaces, both wireless (Wi-Fi and Bluetooth Low Energy) and wired (I2C, SPI, UART). The selected board also has a LoRa communication module, model SEMTECH SX1276 that enables communication at a frequency of 868 MHz.
- Drive: Two 28BYJ-48 stepper motors, powered at 5 V, with 4096 steps per revolution that provide a maximum precision of 0.001534 radians, have been used to drive the two axes of movement of the omnidirectional server presented. The management of the stepper motors requires a controller, for which two units of the type LM298 have been used.
- Communications: Finally, it has been considered that the communications between the position server and the solar trackers require a range according to the typical dimensions of photovoltaic installations. The receiving devices of the orientation command can be arranged in a radius of up to 15 km around the server [61], which is achieved with direct vision between antennas, in optimal conditions, while in unfavourable conditions, such as suburban areas, 3 km are reached [62].

## 3. Results

**Figure 5:**
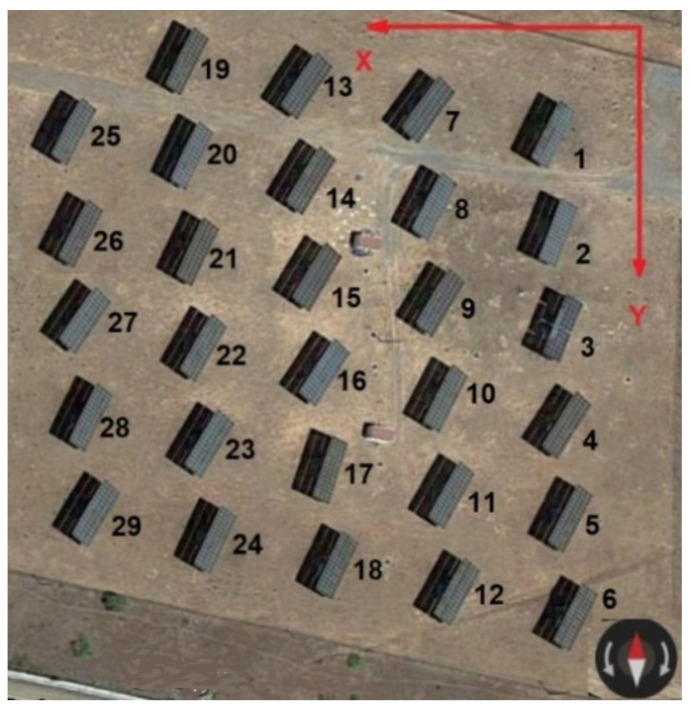
Dual-axis trackers plant Peñarroya I.

This section shows the results obtained when applying this device to the “Peñarroya I” PV plant, situated at a location of 38.299224° N latitude and −5.303114° longitude. The plant consists of 29 dual-axis solar trackers whose collectors measure 12 m wide $\left(a\right)$ by 5 m high $\left(b\right)$. Figure 5 shows its distribution in plan as well as the index assigned to each one and the reference system used to study the system. Table 1 shows the coordinates of the base of each collector.

**Figure 6:**
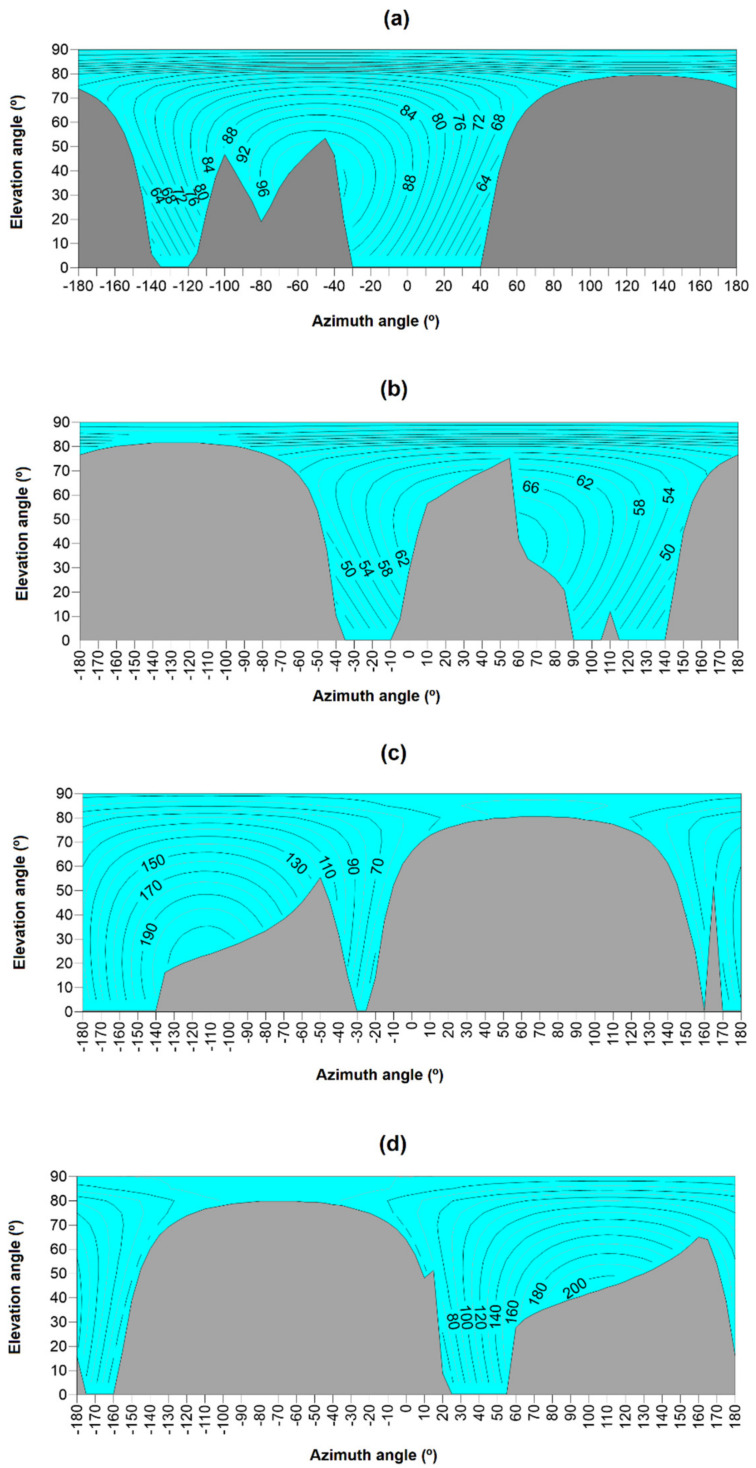
Simulation of the irradiance (W/m^2^) values registered by the proposed device and the fragmentation of the celestial sphere at different moments of time: (**a**) 21 December at 8:24 a.m. in True Solar Time, (**b**) 21 December at 15:24 p.m. in True Solar Time, (**c**) 21 June at 7:30 a.m. in True Solar Time, and (**d**) 21 June at 15:48 p.m. in True Solar Time.

Figure 6 shows the graphic representation of the data obtained by the proposed omnidirectional sensor at different times of the year in Lambert projection hemispheric diagram mode [63]. These figures show the existence of two regions. Thus, the grey region represents the directions of the celestial sphere in which no measurements are taken since it corresponds to positions for which the algorithm prior to tracking indicates inter-shading of collectors. On the other hand, the blue region corresponds to the orientations of the solar servers for which there is no inter-shading and in which, consequently, irradiance (W/m^2^) measurements are made, which are represented by the corresponding iso-level curves (grey lines).

The information shown on each chart is obtained by the sensor during every scanning cycle along the celestial sphere. There is evidence that this is the most complete traceability criterion available in the literature [26,47,64,65]. In general, these methods are limited to the evaluation of installations on flat surfaces, normally horizontal, where only the potential shadows produced by the adjacent collectors are considered. These methods are also limited by the type of tracking they have been developed for. Even for certain types of tracking, such as the tracking of a vertical axis, they have not been computerized due to the lack of a published algorithm [66]. The lack of open-source devices to solve optimal tracking, including backtracking, and of generic algorithms is also evident upon consulting commercial devices to manage backtracking. Although some manufacturers implement algorithms based on artificial intelligence [67,68] or on customized systems [69], authors have not found the theoretical basis of these published.

## 4. Conclusions

The present work shows the construction and design of a device capable of determining the incident solar irradiance on the collector planes of a PV plant with dual-axis trackers depending on their orientation (azimuth and elevation). From this irradiance, obtained by means of instantaneous measurements carried out while tracking the celestial sphere, the device is capable of determining the orientation of the solar trackers for which the incident irradiance on the collectors would be maximum, which allows optimising their energy capture and, consequently, the energy production of the PV plant.

The device described has been developed as Free and open-source hardware (FOSH), which, together with its publication in Open Access, makes it possible for the scientific and/or technological community to access all the details and therefore be able to analyse, modify or improve its design. Thus, it is presented as a pioneering technology in the sector as it is a solution that is operational but simultaneously open to improvement by the scientific community in the framework of collaborative scientific-technical projects, assuming a revolution in the progress of science and technology.

Furthermore, as a novelty, in this device an ex professo algorithm has been implemented to discriminate at all times those celestial orientations that would imply inter-shading between the collectors of the PV plant. To do this, the device integrates the implementation of tracking and backtracking methodologies characterised and simulated by the authors [39,40] (Fernández-Ahumada et al., 2020b, 2020a) in different photovoltaic plants under irradiance conditions described by empirical models. In this way, the solar trackers do not have to calculate the solar position using astronomical algorithms while taking into account other factors that also affect the incident solar irradiance, such as cloud cover, inter-shading between collectors, etc.

According to the aforementioned, the authors consider that the implementation of this device in photovoltaic plants will make it possible to improve the production of the PV plants while managers will be able to have real information both in terms of collectors and in other alternatives.

## Figures and Tables

**Table 1:** Coordinates (m) considered for each tracker.

Tracker	x (m)	y (m)	z (m)
1	18.50	22.70	0.00
2	22.29	40.70	0.00
3	26.08	58.71	0.00
4	29.88	76.71	0.00
5	33.67	94.72	0.00
6	37.46	112.72	0.00
7	41.50	17.86	0.00
8	45.29	35.86	0.00
9	49.08	53.87	0.00
10	52.87	71.87	0.00
11	56.66	89.88	0.00
12	60.46	107.88	0.00
13	64.49	13.01	0.00
14	68.28	31.02	0.00
15	72.08	49.02	0.00
16	75.87	67.03	0.00
17	79.66	85.03	0.00
18	83.45	103.04	0.00
19	87.49	8.17	0.00
20	91.28	26.17	0.00
21	95.07	44.18	0.00
22	98.86	62.18	0.00
23	102.66	80.19	0.00
24	106.45	98.19	0.00
25	114.27	21.33	0.00
26	118.07	39.34	0.00
27	121.86	57.34	0.00
28	125.65	75.35	0.00
29	129.44	93.35	0.00

## Data Availability

Data sharing not applicable.

## Glossary

a	solar collector width
b	solar collector height
$d_{j}$	Julian day
i	index assigned to each solar tracker
j	secondary index assigned to each solar tracker (i≠j)
N	number of solar trackers in the installation
$\vec{i},\vec{j},\vec{k}$	unit vectors associated to a local Cartesian system
$\vec{s}$	solar vector
$s_{x},\;s_{y},\;s_{z}$	components of solar vector
$\vec{n}$	unit vector perpendicular to the collector surface that indicates the direction towardsthe tracker is oriented
$\vec{u}$	unit horizontal vector included in the collector plane
$\vec{v}$	unit vector included in the collector plane and perpendicular to $\vec{u}$
t	specific solar hour
x, y, z	Cartesian coordinates of the base of each solar tracker
$x\; [i],\;y [i],\;z [i]$	arrays containing information about coordinates of each solar tracker
**Greek Letters**	
α	elevation angle of the collector
γ	azimuth angle of the collector rotation axis
φ	latitude
Ω	Earth’s rotation speed
δ	solar declination
θ	angle of incidence of sunbeams on the inclined plane
Γ	auxiliary angular magnitude dependent on the Julian day
